# Defining Coronary Flow Patterns: Comprehensive Automation of Transthoracic Doppler Coronary Blood Flow

**DOI:** 10.1038/s41598-018-35572-4

**Published:** 2018-11-22

**Authors:** Ian L. Sunyecz, Patricia E. McCallinhart, Kishan U. Patel, Michael R. McDermott, Aaron J. Trask

**Affiliations:** 1 Center for Cardiovascular Research, The Research Institute at Nationwide Children’s Hospital, Columbus, OH USA; 2 Department of Pediatrics, The Ohio State University College of Medicine, Columbus, OH USA

## Abstract

The coronary microcirculation (CM) plays a critical role in the regulation of blood flow and nutrient exchange to support the viability of the heart. In many disease states, the CM becomes structurally and functionally impaired, and transthoracic Doppler echocardiography can be used as a non-invasive surrogate to assess CM disease. Analysis of Doppler echocardiography is prone to user bias and can be laborious, especially if additional parameters are collected. We hypothesized that we could develop a MATLAB algorithm to automatically analyze clinically-relevant and non-traditional parameters from murine PW Doppler coronary flow patterns that would reduce intra- and inter-operator bias, and analysis time. Our results show a significant reduction in intra- and inter-observer variability as well as a 30 fold decrease in analysis time with the automated program vs. manual analysis. Finally, we demonstrated good agreement between automated and manual analysis for clinically-relevant parameters under baseline and hyperemic conditions. Resulting coronary flow velocity reserve calculations were also found to be in good agreement. We present a MATLAB algorithm that is user friendly and robust in defining and measuring Doppler coronary flow pattern parameters for more efficient and potentially more insightful analysis assessed via Doppler echocardiography.

## Introduction

The coronary microcirculation (CM) is unique in physiologic structure and function compared to other microvascular beds. It plays a critical role in the regulation of blood flow and nutrient exchange to support the viability of the heart. Because this vasculature lies within the surrounding myocardium, it constantly experiences transmural forces in the form of systolic contraction and diastolic relaxation with every cardiac cycle. This dictates the amount of blood flow regulation to a large extent, in conjunction with the CM’s response to neural and hormonal factors. Compared to other blood flow in the body, coronary flow is unique in that it primarily occurs during myocardial diastole, whereas the CM is mostly occluded during systole^1^.

Transthoracic Doppler Echocardiography (TTDE) has proven to be a useful and relatively inexpensive tool to non-invasively assess cardiac perfusion by measuring parameters such as coronary flow velocity reserve (CFVR) and velocity-time integral (VTI)^2–6^. For both human and animal subjects, blood flow is measured via one of the major coronary arteries (left main, right main, left anterior descending) at various windows under both baseline and stress conditions, which yields the characteristic biphasic coronary flow pattern (CFP). In many disease states however, the CM becomes structurally and functionally impaired leading to changes in TTDE measurements and can thus be indicative of progressing CM pathology.

For example, in adult humans with type II diabetes mellitus (T2DM), multiple studies have demonstrated a reduction in coronary flow reserve (CFR) or CFVR compared to matched controls despite being asymptomatic^7–9^. Previous studies by our laboratory have shown that the CM in young T2DM murine and porcine models undergoes early inward hypertrophic remodeling^10,11^, and others have demonstrated functional deficits^12,13^. Structural remodeling was associated *in vivo* with a reduction in coronary blood flow (CBF) and CFR. Moreover, this remodeling occurred prior to occlusive macrovascular atherosclerosis which further suggests the importance of examining the CM especially early in disease progression^11^. Of additional interest, recent studies have shown significant differences in systolic and/or diastolic portions of these unique biphasic CFPs between normal and diabetic patients, suggesting that additional parameters obtained from coronary TTDE may be useful in clinical diagnostics^14,15^.

A disadvantage to TTDE is that manual analysis by a clinician or technician is still required to assess these perfusion parameters, which is often a time consuming process. Collecting additional parameters from these CFPs would only elongate the laborious process. The guidelines set forth by the American Society of Echocardiography state that measurements from only 3–4 cardiac cycles deemed representative by the rater are sufficient to obtain an average, yet multiple studies have shown significant differences between intra- and inter-operator variability in manual TTDE analysis^16–19^. Creating an automated or nearly automated algorithm to analyze CFPs would reduce user bias, allow for the analysis of many more cardiac cycles, and reduce manual labor.

In this study, we aimed to develop a MATLAB program that would (1) automatically extract existing and clinically utilized parameters of murine coronary TTDE flow patterns such as peak velocity (PV), heart rate (HR), and VTI as well as newly-defined times, velocities, and slopes, from raw exported video files and (2) automatically perform calculations to obtain parameter averages and CFVR per animal. We hypothesized that this program would significantly reduce analysis time compared to manual analysis. Additionally, we hypothesized that the measurements obtained from the program would be in good agreement with manual analysis and that the program would reduce inter and intra-operator variability.

## Materials and Methods

### Animals

Our lab had previously conducted multiple studies on 16- and 24-wk male homozygous (db/db) diabetic and age-matched heterozygote (Db/db) non-diabetic mice from The Jackson Laboratories. 3 db/db and 3 Db/db mice were randomly selected from these previous studies for inclusion into the current study to compare manual vs. automated analysis. Additionally, we selected 18 Db/db and 20 db/db 16-wk mice to undergo analysis solely with our program to determine if our newly selected parameters showed any differences based on disease. Mice were housed under a 12-hr light/dark cycle at 22 °C and 60% humidity. They were allowed *ad libitum* access to water and were fed standard laboratory mouse chow. This study was conducted in accordance with National Institutes of Health Guidelines and was approved by the Institutional Animal Care and Use Committee at The Research Institute at Nationwide Children’s Hospital.

### Transthoracic Doppler Echocardiography

Coronary blood flow velocity was measured noninvasively with a high-frequency, high-resolution ultrasound unit (Vevo2100, Visual Sonics, Toronto, Canada) equipped with a 30 MHz probe, at baseline (1% isoflurane), and under conditions of maximum flow (hyperemia, 3% isoflurane) as previously described^10^. Doppler measurements of the left main coronary artery diameter and flow were performed under a modified four chamber view. Mice were anesthetized with 2% isoflurane vaporized with 100% oxygen. Following induction, isoflurane was reduced to 1% to determine baseline coronary flow, and then increased to 3% to measure maximal coronary flow. Baseline and hyperemic PW Doppler files were either manually analyzed on the Vevo 2100 software or exported for automated analysis using the MATLAB program.

### Coronary Flow Pattern Program

The CFP program was developed using MATLAB software (The MathWorks Inc., Natick, MA) and was designed to analyze and extract time intervals, velocity points, and slopes from PW Doppler CFP AVI video files exported from Vevo 2100 software. A detailed description of our algorithm for processing the video files and extracting the desired parameters can be found in the online supplemental section.

#### Data Exportation

Raw AVI video files were directly exported from the Vevo2100 software for analysis. Specifically, uncompressed PW Doppler AVI video files were automatically exported at a size of 880 × 666 pixels, a frame rate of 30 frames per second, and sweep speed parameter of 0.85 seconds.

#### Region of Interest Extraction

**Figure 1:**
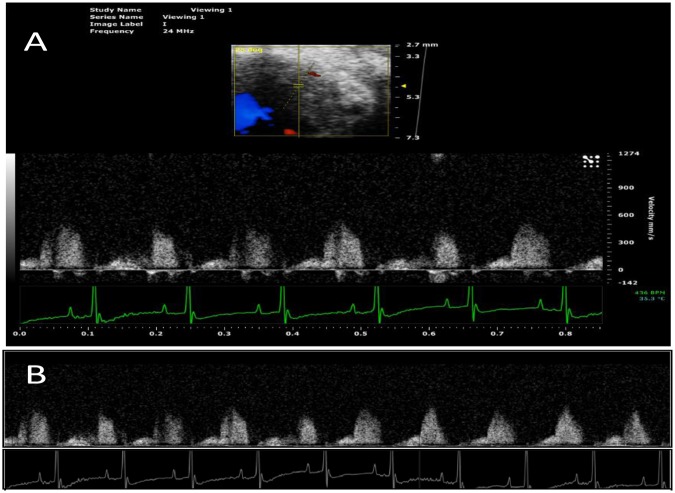
(**A**) Example PW Doppler coronary flow pattern file exported from Vevo2100 software. Exported as is, these files contain the desired coronary Doppler window region as well as a time axis, a velocity axis, an ECG recording, a B-Mode window, and various study labels. (**B**) Full PW Doppler and ECG sequence parsed together from the single recording in A with non-pertinent information cropped out.

PW Doppler Files: The raw uncompressed PW Doppler video files contain the desired coronary Doppler window region as well as a time axis, a velocity axis, an ECG recording, a B-Mode window, and various study labels **(**Fig. 1A**)**. In order to extract the complete coronary Doppler region and ECG recording, a number of cropping and parsing steps were initiated and were built upon similar techniques by Magagnin *et al*.^14^ and Zholgharni *et al*.^20^. The final resulting images were the full Doppler CFP sequence from the zero-velocity baseline to the maximum velocity and the full ECG recording **(**Fig. 1B**)**.

#### Feature Extraction and Parameter Measurements

**Figure 2:**
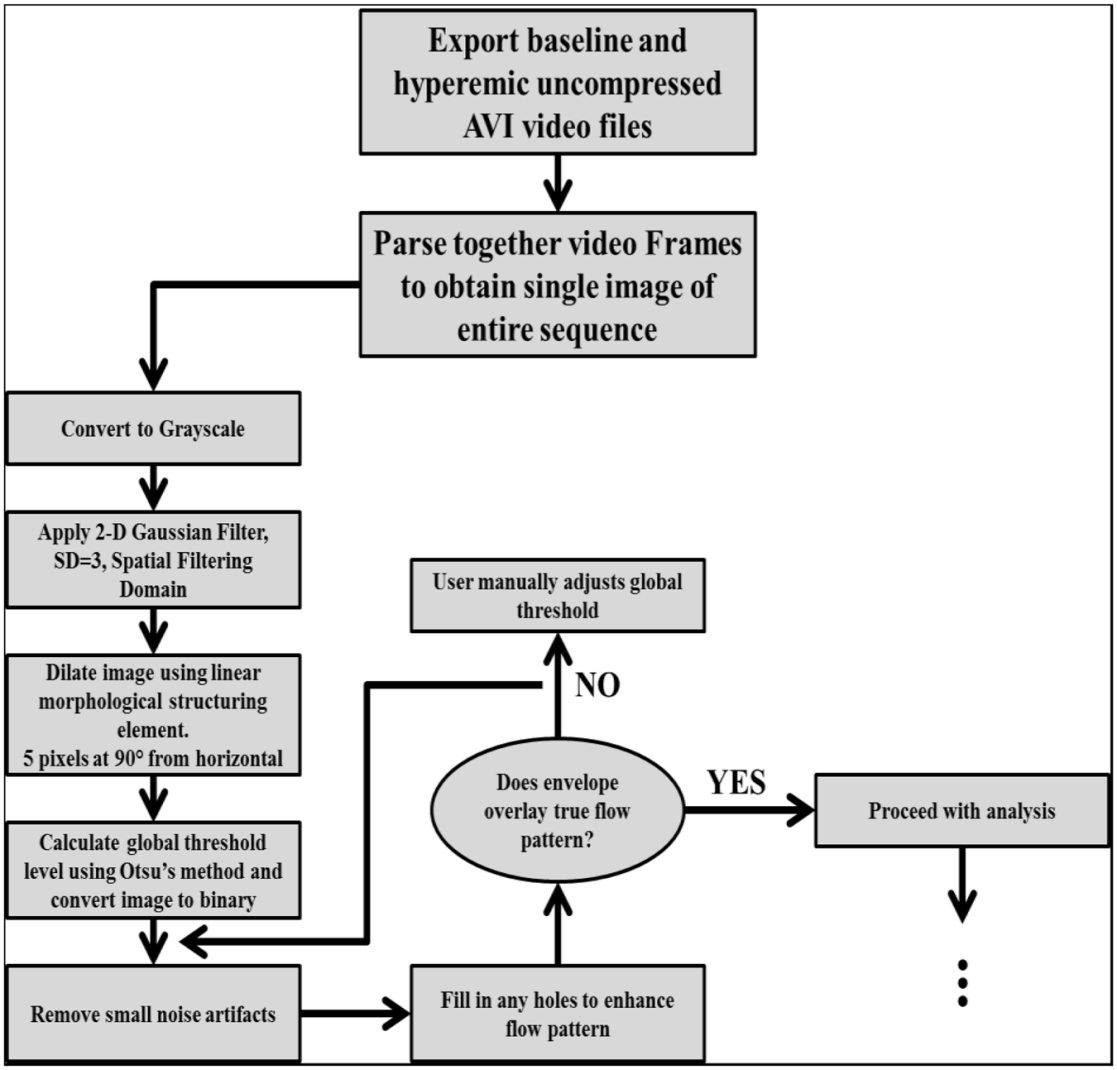
Flow chart of image processing techniques used on the Doppler CFP images.

PW Doppler Files - Envelope Overlay: After cropping the PW Doppler files to obtain both the full CFP sequence and the ECG recording, several image processing techniques were used to create an envelope overlay of the CFP. A flow chart of the image processing techniques is presented in Fig. 2.

**Figure 3:**
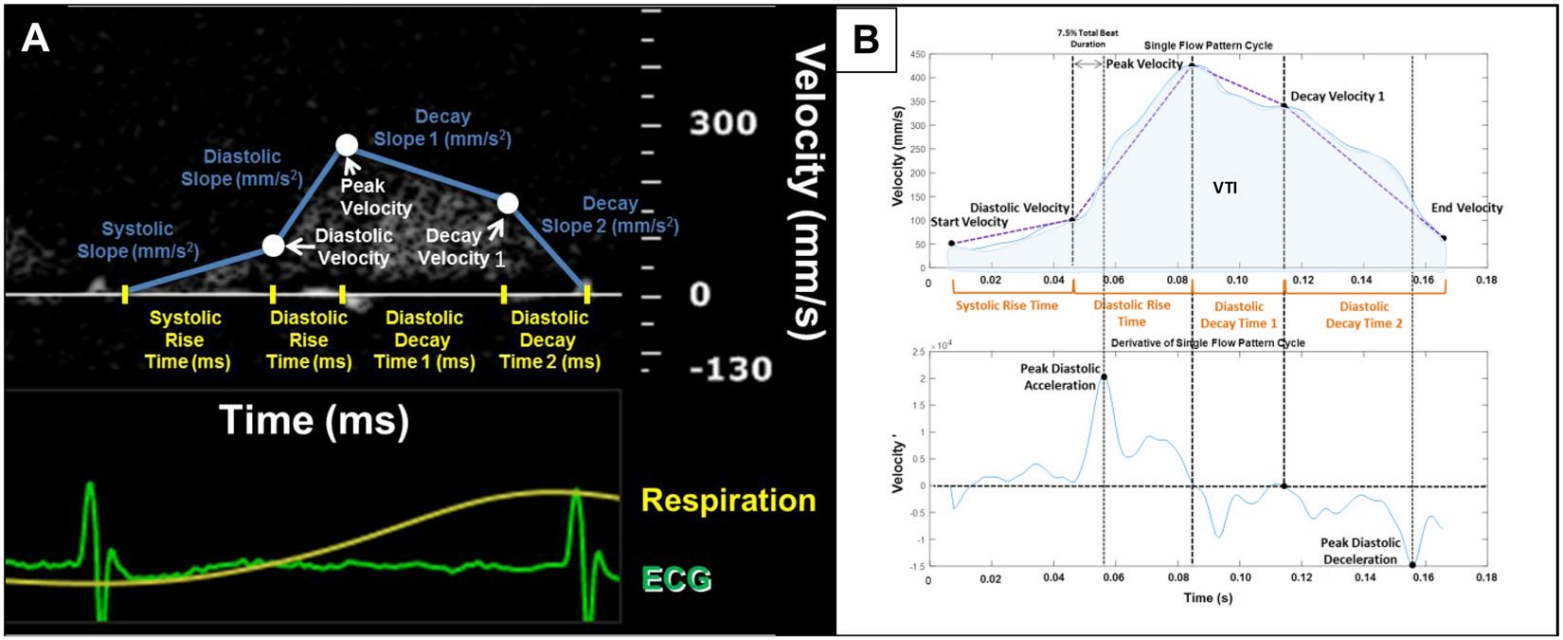
(**A**) Parameters of coronary flow patterns over the course of one cardiac cycle overlaid on Doppler recording. (**B**) Example of a single coronary flow pattern cycle and its corresponding derivative. The velocity and acceleration points (black) were first identified. From there, the time intervals were measured (orange) and the average slopes were calculated (purple).

PW Doppler Files – Parameter Extraction: The ECG recording was used to separate individual CFP cycles per heartbeat. Specifically, the start and end of each CFP cycle was identified as the R-peak to R-peak interval from the ECG. Following separation of each individual CFP cycle, the derivatives of their envelopes were calculated. Both the flow pattern envelope and its derivative were utilized in measuring our newly defined time intervals, velocity points, slopes, VTI and HR specified below (Fig. 3). The parameters were measured and stored for each cycle in the CFP sequence.

- **Peak Velocity** [mm/s]: The peak velocity (PV) was measured as the maximum velocity value for each CFP.
- **Diastolic Velocity** [mm/s]: The diastolic velocity was measured by first identifying the peak diastolic acceleration (PDA) of the CFP derivative. A window of 7.5% of the total beat duration was calculated and the diastolic velocity was chosen as the minimum value within that window up until the PDA.
- **Decay Velocity 1** [mm/s]: The decay velocity 1 was measured by first identifying the peak diastolic deceleration (PDD) of the CFP derivative. Between the PV and the PDD, decay velocity 1 was selected as the point at which the acceleration crossed the x-axis closest to the PDD.
- **Systolic Rise Time** [ms]: The systolic rise time was measured as the time interval between the start of the CFP to the diastolic velocity.
- **Diastolic Rise Time** [ms]: The diastolic rise time was measured as the time interval between the diastolic velocity to the PV.
- **Diastolic Decay Time 1** [ms]: The diastolic decay time 1 was measured as the time interval between the PV to the decay velocity 1.
- **Diastolic Decay Time 2** [ms]: The diastolic decay time 2 was measured as the time interval between the decay velocity 1 to the end of the CFP cycle.
- **Systolic Slope** [mm/s^2^]: The systolic slope was calculated as the average slope from the start of the CFP cycle to the diastolic velocity.
- **Diastolic Slope** [mm/s^2^]: The diastolic slope was calculated as the average slope from the diastolic velocity to the PV.
- **Decay Slope 1** [mm/s^2^]: The decay slope 1 was calculated as the average slope from the PV to the decay velocity 1.
- **Decay Slope 2** [mm/s^2^]: The decay slope 2 was calculated as the average slope from the decay velocity 1 to the end of the CFP.
- **Heart Rate** [BPM]: The heart rate (HR) was measured as the time duration of the R-R interval.
- **Velocity Time Integral** [mm]: The velocity time integral (VTI) was measured by integrating the CFP cycle envelope.

**Figure 4:**
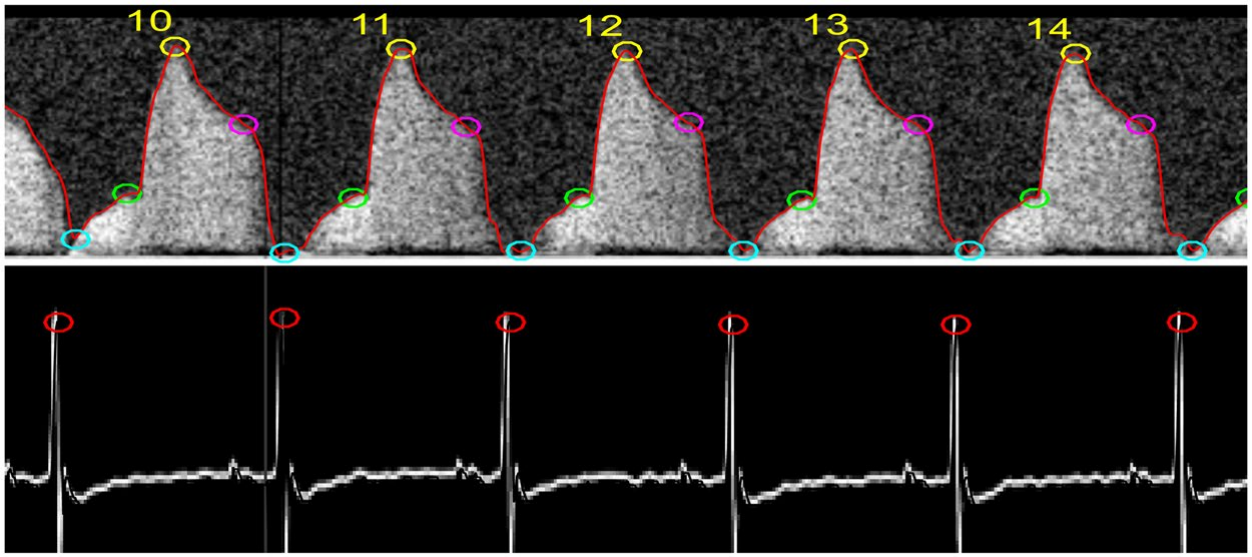
An ideal CFP paired with its corresponding ECG analyzed using the MATLAB program. The circles indicate where the program identified the velocity points. The numbers indicate the cycle count from the beginning of the sequence.

In addition to extracting the specified parameters from PW Doppler files, the program averages each parameter per animal and calculates CFVR. Often in TTDE, certain cycles should be excluded from analysis, as they are not representative of cycles in the entire sequence (Supplementary Fig. 1). Our MATLAB program also contains a function that eliminates non-representative cycles and outliers from analysis. Figure 4 displays a portion of a CFP analyzed with the program showing the identified velocity points and envelope overlay. Finally, CFVR was calculated by dividing average peak hyperemic velocity into peak baseline velocity: ***CFVR*** = *PVhyperemia/PVbaseline*.

### Validation Protocol

Two trained lab personnel with adequate experience in Doppler CFP analysis performed both manual and program analysis in a blinded manner. One PW Doppler baseline CFP file, and one PW Doppler hyperemic CFP file were analyzed per animal. 6 animals in total were analyzed. The PW Doppler files recordings were 1.20 seconds -4.90 seconds in duration with a minimum of 6 complete cardiac cycles.

#### Manual Analysis Protocol

Each rater analyzed the animal files a total of two times separated by two distinct viewings in a blinded manner. The individual PW Doppler files were blinded and randomized so the rater had no knowledge on the specific animal type or file order. The files were further randomized between viewings. Measurements were obtained in the Vevo 2100 software. Each rater analyzed the same number of CFPs as the start and end cycles were pre labeled prior to analysis. If the rater deemed any of the cycles to be unclear or non-representative, he/she noted that cycle as non-analyzable and continued analysis until the entire sequence was completed. The measurements obtained for each cycle included the 4 time intervals, 5 velocity points, 4 slopes, HR, and VTI. Once flow pattern cycle measurements were made in the Vevo 2100 software, the rater populated a pre-labeled Microsoft Excel datasheet. The total time taken to complete both viewings was recorded.

#### Program Analysis Protocol

The same raters performed the program analysis similarly in two separate viewings. The user ran the MATLAB program for each animal and the measured parameters and calculations were directly exported into a Microsoft Excel datasheet. The total time taken to complete both viewings was recorded as well as time taken to export the raw files into a folder for analysis.

#### Parameter Selection and Disease

After performing the validation study to determine if our program selected parameters similar to a trained rater, we utilized a larger sample size of mice to determine if any of our newly defined parameters differed between normal and disease states as a proof of concept. For this test, we analyzed CFPs with our program from 16-wk Db/db (n = 18) and db/db (n = 20) mice under baseline and stress conditions. The average and standard error of the mean (SEM) was found for each parameter and an unpaired t-test was used to determine if any significant differences were present between normal and diabetic CFP parameters.

#### Statistical/Data Analyses

Intra-observer variability, inter-observer variability, and variability between manual analysis and the MATLAB program were examined. For each of these tests, data was expressed as mean +/− SD. Bland-Altman analysis was executed to calculate bias (mean difference) with limits of agreement set as +/− 2 SD^21^. Linear regression analysis was performed to determine the coefficient of determination (R^2^) and the regression equation. Unpaired t-tests with a significance level of p < 0.05 were also executed. Statistical tests were done in GraphPad Prism 7 software and Microsoft Excel. The CFP program was created in MATLAB and the program analysis protocol was run on a computer utilizing an Intel ® Core ™ i3-2100 CPU @ 3.10 GHz.

## Results

For the PW Doppler files between all 6 mice, there were a total of 98 complete CFP cycles measured at 1% isoflurane (baseline) that could have undergone analysis. There were a total of 117 complete CFPs measured at 3% isoflurane (hyperemia) that could have undergone analysis. Because each rater analyzed the same files in two separate randomized viewings, each frame and CFP had the same chance of being analyzed twice. Supplementary Table 1 specifies the total number of complete CFP cycles that could have undergone analysis for the PW Doppler files separated by animal. PV, HR, and VTI results under baseline and hyperemic conditions are presented below as they are the most frequently measured in clinical practice.

### Intra-Rater Variability

To determine the intra-rater variability for manual analysis and for the program analysis, only CFPs that were analyzed in both viewings per rater were included. If a CFP was analyzed in only one viewing, or not analyzed in either viewing, the cycle was excluded. This was deemed appropriate as the twice analyzed cycles were likely the cleanest and most representative cycles. Supplementary Fig. 2 displays representative images of both the manual analysis and the program analysis over the same set of cycles to highlight the variability between viewings. Bland-Altman and linear regression analysis were performed on each rater’s consistent cycles for PV, HR, and VTI. Table 1 shows the intra-rater variability of these parameters for both manual analysis and the program analysis.

**Table 1:** Intra-rater variability between manual and automated analysis for both raters. Bland-Altman analysis and linear regression analysis were performed for each parameter.

		Rater 1 Manual				Rater 1 Automated			
		Bias	+/− 2 SD	Regression Equation	R^2^	Bias	+/− 2 SD	Regression Equation	R^2^
Baseline	PV (mm/s)	−5.668	41.340	y = 0.947x + 20.58	0.984	−0.685	17.930	y = 1.047x − 11.28	0.997
	HR (BPM)	−0.070	1.570	y = 1.003x − 1.122	0.999	0	0	y = x	1
	VTI (mm)	−0.282	3.874	y = 0.986x + 0.555	0.968	0.120	3.028	y = 1.023x − 0.421	0.995
Stress	PV (mm/s)	6.070	61.940	y = 0.952x + 28.93	0.984	4.273	33.500	y = 1.02x − 19.3	0.995
	HR (BPM)	−0.080	1.900	y = x + 0.042	0.999	0	0	y = x	1
	VTI (mm)	1.910	6.600	y = 1.052x − 4.73	0.928	0.834	1.950	y = 1.012x − 1.374	0.993
		**Rater 2 Manual**				**Rater 2 Automated**			
		**Bias**	**+/− 2 SD**	**Regression Equation**	**R** ^**2**^	**Bias**	**+/− 2 SD**	**Regression Equation**	**R** ^**2**^
Baseline	PV (mm/s)	22.460	45.120	y = 0.945x − 4.767	0.965	−0.246	6.704	y = 1.014x − 3.35	0.999
	HR (BPM)	0.090	4.000	y = 1.003x − 1.079	0.999	0	0	y = x	1
	VTI (mm)	0.773	4.518	y = 0.907x + 1.348	0.923	0.008	1.038	y = 1.023x − 0.4207	0.995
Stress	PV (mm/s)	13.040	72.820	y = 0.994x − 8.76	0.975	−5.306	45.360	y = 1.037x − 22.46	0.992
	HR (BPM)	−0.300	3.600	y = 1.003x − 0.8177	0.999	0	0	y = x	1
	VTI (mm)	2.865	7.194	y = 0.825x + 6.42	0.932	−0.430	3.804	y = 1.081x − 3.351	0.981

PV, VTI, and HR intra-rater variability was reduced in the program analysis for both raters as evidenced by a bias nearing closer to 0 and smaller limits of agreement in the Bland-Altman analysis. Additionally, the coefficient of determination neared closer to 1 in the linear regression analysis. HR variability in the program analysis was nullified completely as this section of the program is fully automated. The decreasing trend in variability with the program was found in both baseline and hyperemic CFPs for the listed parameters.

### Inter-Rater Variability

Supplementary Tablesnual and program inter-rater variability, only cycles that were analyzed in both viewings and that were consistent among each rater were included. As these CFPs were analyzed twice by both raters, they were deemed appropriate to incorporate into this analysis. Average values between the two viewings were calculated for each rater and the total number of cycles to determine the inter-rater variability is shown in Supplementary Table 2. Bland-Altman and linear regression analysis were performed on the rater’s averages for their consistent cycles for PV, HR, and VTI. Table 2 displays the statistical figures for the inter-rater variability.

**Table 2:** Inter-rater variability between manual and automated analysis. Bland-Altman analysis and linear regression analysis were performed for each parameter.

		Manual				Automated			
		Bias	+/−2SD	Regression Equation	R^2^	Bias	+/−2SD	Regression Equation	R^2^
Baseline	PV (mm/s)	−3.971	47.78	y = 1.09x − 25.65	0.987	2.197	5.962	y = 0.981x + 2.869	0.999
	HR (BPM)	−0.222	2.68	y = 1.01x − 1.745	0.998	0	0	y = x	1
	VTI (mm)	−1.492	5.72	y = 1.113x − 0.951	0.951	0.423	1.21	y = 0.971 + 0.01	0.994
Stress	PV (mm/s)	−13.48	54.1	y = 1.062x − 32.31	0.991	9.239	28.62	y = 0.993x − 3.745	0.996
	HR (BPM)	−0.07	2.64	y = 0.994x − 2.372	0.999	0	0	y = x	1
	VTI (mm)	−4.68	8.19	y = 1.129x − 1.79	0.889	1.137	2.012	y = 0.9507x + 1.191	0.995

Variability between raters was reduced when using the program to analyze CFPs for PV, HR, and VTI at 1% and 3% isoflurane. This is supported by the bias’s tending closer to 0 and smaller limits of agreement in the Bland-Altman analysis as well as larger coefficients of determination in the linear regression analysis.

### Manual Analysis vs. MATLAB Program Analysis

While the MATLAB program reduced variability individually and between different raters, it was equally important that the program selected parameters that were in good agreement with manual analysis. The program was tested to assess its ability in finding total parameter averages per animal.

#### Animal by Animal Validation

**Figure 5:**
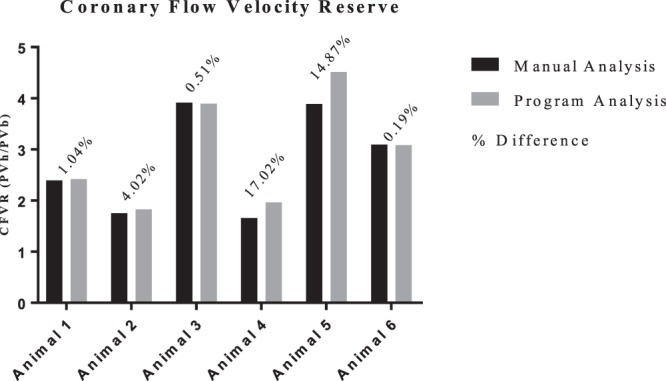
CFVR per animal comparing between the program analysis vs. the manual analysis. The overlaying percentages shows the percent difference between the two methods per animal.

Often, parameters are averaged across a number of flow patterns for each subject/animal in order to obtain representative measurements. The purpose of this validation was to compare the average parameter output of the program to the manual average for each mouse. For each CFP per animal at 1% and 3% isoflurane, the mean PV, VTI, and HR were calculated by averaging both raters’ measurements. If a cycle was not analyzed by either rater, that cycle was excluded (Supplementary Table 3). The final average and SD for each animal was then calculated as the average of the analyzed cycles. The same process was repeated for the program analysis. If the program found a cycle to be “not-representative” via the exclusion algorithm, it was not included in the analysis. Table 3 shows the results between the manual and program analysis as well as the percent difference between the two. Percent differences larger than 10% are bolded. Figure 5 shows the comparison of CFVR per animal between the manual analysis and program analysis. Average HR was equal to or less than 1% different between the two methods. There was a significant difference in both PV and VTI for some animals. Specifically, 3/6 baseline CFP files and 2/6 hyperemic CFP files showed a significant difference in average PV between manual analysis and the program analysis. However, the percent difference between the two methods for these animals was no greater than 17.43%. 4/6 baseline flow patterns and 2/6 hyperemic flow patterns showed a significant difference in average VTI with the largest percent difference of 55.07% found in one animal. The greatest percent difference in the CFVR calculation was 17.02% in one animal while 4/6 animals showed a percent difference of less than 5% between the two methods.

**Table 3:** Comparison between the program and manual analysis for average measurements per animal.

1%-Baseline	Peak Velocity (mm/s)							
	Manual Analysis			Program Analysis			p-value	% Difference
	Average	SD	n	Average	SD	n		
Animal 1	248.728	47.256	9	227.161	52.189	9	0.372	9.06
Animal 2	533.739	35.025	10	510.590	47.521	8	0.251	4.43
Animal 3	260.946	19.825	13	257.125	27.249	9	0.707	1.47
Animal 4	241.672	27.386	19	202.918	25.345	14	<0.05	**17.43**
Animal 5	165.464	13.603	16	141.567	11.004	11	<0.05	**15.57**
Animal 6	351.239	22.818	15	311.908	32.140	18	<0.05	**11.86**
**3%-Hyperemic**	**Manual Analysis**			**Program Analysis**			**p-value**	**% Difference**
	**Average**	**SD**	**n**	**Average**	**SD**	**n**		
Animal 1	595.107	37.393	11	549.276	38.318	11	<0.05	8.01
Animal 2	935.917	20.741	6	932.475	26.316	6	0.806	0.37
Animal 3	1021.542	20.166	17	1001.462	48.625	16	0.127	1.99
Animal 4	400.134	27.892	13	398.618	11.846	9	0.88	0.38
Animal 5	643.827	44.016	27	639.422	53.611	23	0.751	0.69
Animal 6	1086.068	32.154	15	962.623	110.616	15	<0.05	**12.05**
**1%-Baseline**	**Heart Rate (BPM)**							
	**Manual Analysis**			**Program Analysis**			**p-value**	**% Difference**
	**Average**	**SD**	**n**	**Average**	**SD**	**n**		
Animal 1	343	13	9	342	13	9	0.872	0.29
Animal 2	435	1	10	433	2	8	<0.05	0.46
Animal 3	402	7	13	402	6	9	>0.99	0.00
Animal 4	313	16	19	316	17	14	0.608	0.95
Animal 5	397	4	16	396	5	11	0.569	0.25
Animal 6	473	1	15	471	2	18	<0.05	0.42
**3%-Hyperemic**	**Manual Analysis**			**Program Analysis**			**p-value**	**% Difference**
	**Average**	**SD**	**n**	**Average**	**SD**	**n**		
Animal 1	375	2	11	373	3	11	0.081	0.53
Animal 2	427	2	6	425	3	6	0.204	0.47
Animal 3	464	5	17	460	6	16	<0.05	0.87
Animal 4	298	13	13	295	10	9	0.567	1.01
Animal 5	357	8	27	356	9	23	0.679	0.28
Animal 6	390	4	15	390	8	15	> 0.99	0.00
**1%-Baseline**	**VTI (mm)**							
	**Manual Analysis**			**Program Analysis**			**p-value**	**% Difference**
	**Average**	**SD**	**n**	**Average**	**SD**	**n**		
Animal 1	19.205	3.308	9	15.071	1.792	9	<0.05	**24.12**
Animal 2	35.822	2.010	10	33.491	2.653	8	<0.05	6.73
Animal 3	19.054	1.134	13	17.058	0.746	9	<0.05	**11.06**
Animal 4	24.857	2.715	19	20.039	3.648	14	<0.05	**21.46**
Animal 5	10.130	0.981	16	9.378	1.244	11	0.091	7.71
Animal 6	20.201	2.224	15	16.290	2.700	18	<0.05	**21.44**
**3%-Hyperemic**	**Manual Analysis**			**Program Analysis**			**p-value**	**% Difference**
	**Average**	**SD**	**n**	**Average**	**SD**	**n**		
Animal 1	46.055	3.118	11	32.673	4.684	11	<0.05	**34.00**
Animal 2	60.471	1.691	6	55.757	2.026	6	<0.05	8.11
Animal 3	62.597	2.448	17	59.229	3.045	16	<0.05	5.53
Animal 4	33.220	3.237	13	31.584	1.371	9	0.1705	5.05
Animal 5	50.719	4.029	27	50.171	5.776	23	0.696	1.08
Animal 6	78.245	10.525	15	44.459	15.516	15	<0.05	**55.07**

#### Analysis Duration

Though there were only 6 animals included in this validation study, the manual analysis portion was time and labor intensive mainly because 13 total measurements were made for each analyzed PW Doppler cycle (only three clinically-relevant measures are reported here). On average, it took the raters approximately 1500 total minutes to complete the manual analysis of both viewings. For the program analysis, the raters spent approximately 50 total minutes completing both viewings including time taken to export the raw video files. That is a 30-fold decrease in time spent when using the MATLAB program to measure the specified CFP parameters over manual intervention.

#### Parameter Values Between Normal and Disease

We show an appreciable agreement between our program and manual analysis when identifying clinically useful parameters of coronary flow. Additionally, we aimed to determine whether our newly identified parameters varied between normal and disease. Table 4 lists the average and SEM for each parameter as well as the p-value when comparing normal and diabetic mice. There were a number of significant differences especially under hyperemic conditions. All velocity values and HR were significantly reduced in diabetic mice under stress. Decay slopes 1 and 2 were significantly decreased while a significant increase was noted in diastolic decay time 1 for diabetic mice under hyperemic conditions. The only parameter significantly different under normal conditions was HR.

**Table 4:** Comparison of newly defined parameters between normal and diabetic mice under baseline and hyperemic conditions from program analysis.

	Group	Db/db (n = 18)	SEM	db/db (n = 20)	SEM	p-value
Baseline	Systolic Rise Time (ms)	**53.635**	3.864	**56.963**	2.236	0.4498
	Diastolic Rise Time (ms)	**26.871**	1.271	**28.842**	1.156	0.2579
	Diastolic Decay Time 1 (ms)	**28.283**	1.967	**34.200**	2.844	0.1027
	Diastolic Decay Time 2 (ms)	**32.729**	1.855	**31.806**	2.187	0.7521
	Systolic Slope (mm/s^2^)	**499.910**	135.667	**205.930**	104.684	0.0914
	Diastolic Slope (mm/s^2^)	**8334.587**	619.389	**7832.198**	545.271	0.5449
	Decay Slope 1 (mm/s^2^)	**−3399.582**	339.016	**−2727.118**	290.579	0.1387
	Decay Slope 2 (mm/s^2^)	**−5007.732**	497.366	**−5257.814**	513.631	0.7297
	Diastolic Velocity (mm/s)	**110.423**	29.403	**86.978**	25.820	0.5512
	Peak Velocity (mm/s)	**315.927**	30.750	**295.421**	23.718	0.5967
	Decay Velocity 1 (mm/s)	**227.881**	29.243	**212.113**	26.724	0.6924
	Heart Rate (BPM)	**432**	8	**396**	5	**0.0007**
	VTI (mm)	**24.556**	4.623	**23.581**	4.243	0.8772
Hyperemia	Systolic Rise Time (ms)	**53.513**	1.685	**50.623**	1.351	0.1853
	Diastolic Rise Time (ms)	**26.606**	0.796	**26.780**	0.741	0.8733
	Diastolic Decay Time 1 (ms)	**22.931**	1.995	**33.767**	1.391	**0.0001**
	Diastolic Decay Time 2 (ms)	**30.359**	2.190	**32.813**	1.569	0.3614
	Systolic Slope (mm/s^2^)	**4907.218**	535.500	**3563.728**	287.326	**0.0291**
	Diastolic Slope (mm/s^2^)	**18976.735**	1166.904	**17787.362**	1002.266	0.4420
	Decay Slope 1 (mm/s^2^)	**−11830.073**	1401.289	**−7289.421**	537.252	**0.0033**
	Decay Slope 2 (mm/s^2^)	**−18396.149**	1125.565	**−13422.726**	857.346	**0.0011**
	Diastolic Velocity (mm/s)	**363.269**	35.752	**240.222**	21.748	**0.0048**
	Peak Velocity (mm/s)	**846.456**	39.760	**693.027**	28.672	**0.0031**
	Decay Velocity 1 (mm/s)	**613.977**	36.966	**459.309**	24.555	**0.0011**
	Heart Rate (BPM)	**452**	6	**418**	6	**0.0004**
	VTI (mm)	**55.348**	4.377	**48.879**	3.483	0.2506

## Discussion

The CM is a unique and important microvascular bed that supports the viability of the heart through blood flow and nutrient exchange while constantly experiencing transmural contraction and relaxation forces. In the progression of certain diseases, the CM has been shown to become structurally and/or functionally impaired, often prior to occlusive macrovascular atherosclerosis, which can lead to decreased perfusion, ischemic events, and myocardial infarction. TTDE is a non-invasive clinical tool that has been successfully utilized to assess these impairments of blood flow regulation in the CM. Analysis of coronary TTDE examinations however can be time consuming and prone to operator measurement variability. Automating this analysis could reduce operator variability, allow for the incorporation of more cardiac cycles with additional parameters measured, and reduce labor time. There are, to date, a limited number of studies concerning the automation of TTDE CFP analysis and none, to our knowledge, have been published utilizing an echocardiographic software intended for animal models.

We aimed to develop a MATLAB program that could analyze PW Doppler CFP files exported directly from our Visual Sonics Doppler Echocardiography machine system to (1) automatically extract VTI, HR, and newly defined time intervals, velocities, and average slopes from murine CFPs and (2) automatically calculate CFVR. Finally, we hypothesized that this program would significantly reduce analysis time, that measurements obtained from the program would be in good agreement with manual analysis, and that the program would reduce inter and intra-operator variability.

Our results show that our MATLAB program was able to effectively reduce intra- and inter-operator variability when selecting PV, HR, and VTI parameters for analysis. For example, average PV difference between manual viewings was as high as 22 mm/s and difference between raters as high as 13 mm/s. Our MATLAB program, reduced this variability in mean difference to as low as 5 mm/s between manual viewings and 9 mm/s between raters respectively. Reducing variability in TTDE analysis individually and between operators is of critical importance to accurately and precisely make conclusions about blood flow and the CM.

We then investigated how well our MATLAB program agreed with manual analysis in parameter selection by comparing the averages from each animal. While the percent difference was minimal in mean HR for all animals, there were some mean significant differences in PV and VTI. Generally, the program measurements for PV were closer to the manual measurements under hyperemic conditions, with only 2/6 animals being significantly different. Contrarily, 3/6 animals were significantly different for PV when evaluated under baseline conditions. Under 3% isoflurane administration, the coronary arteries dilate resulting in larger blood flow velocity profiles for each cardiac cycle. Thus, the PW Doppler flow patterns often become fuller and more distinct, which is likely why the program better selected average PVs per animal under hyperemic conditions.

However, the largest percent difference found for PV in this validation under both 1% and 3% isoflurane was 17.43% (Animal 4, 1% isoflurane), which corresponded to an average difference of approximately 39 mm/s for that animal. The Vevo 2100 software, given the window size that manual PV measurements were made, is accurate to approximately 4–5 mm/s per pixel for the velocity axis. The 39 mm/s difference measured between the program and manual analysis for that animal therefore was only a difference of ~9 pixels. As shown above in the Bland-Altman limits of agreement, the intra-observer variability for PV was larger than 39 mm/s for the raters. Despite the program significantly under-estimating PV in some animals, the deviation was small on a pixel scale and the largest deviation was still found to be less than the intra-observer error.

**Figure 6:**
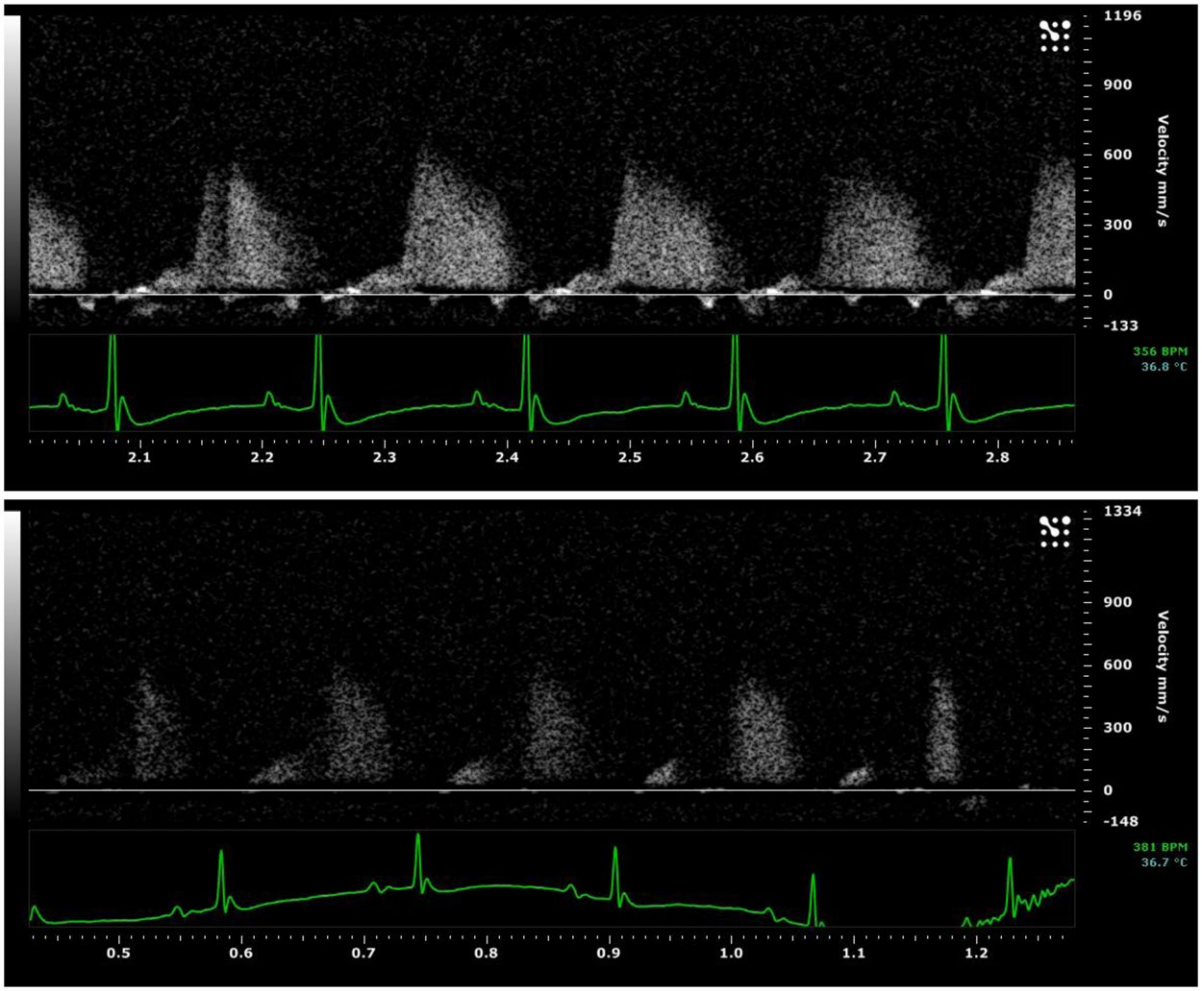
Images of two separate PW Doppler CFP recordings. The complete and expected biphasic pattern is present in the top window compared to the bottom window where a portion of the biphasic pattern is shadowed.

It is important to note the degree of variability that is present in TTDE recordings, especially with murine coronary flow. Often, the expected biphasic outline observed in coronary TTDE can be partially shadowed making parameter extraction difficult and subjective, even with manual interpretation (Fig. 6). In situations where CFP cycles were partially shadowed, we found that the raters would often extrapolate the CFP envelope to create the expected biphasic outline, basing their outline on flow patterns before and after the cycle in question or from previous experience. This invariably led to the measurement differences observed between our program and the manual analysis.

VTI was also found to be significantly different in some animals. Similar to PV, VTI was more accurately measured by the program under hyperemic conditions because the flow patterns were fuller and more distinct. Under baseline conditions, 5/6 animals VTI was greater than 10% different between the two analysis methods. This was also a result of manual extrapolation leading to an over-estimation of VTI. The largest percent difference was found to be 55.07% which corresponds to an average difference of ~34 mm (Animal 6, 3% isoflurane). While recordings with clearer and more distinct biphasic CFPs would have certainly validated more closely in this animal to animal comparison, CFPs that require extensive extrapolation may not be suitable for the program. To our knowledge, there is no criteria for determining when to exclude a CFP from analysis when the full biphasic pattern is partially shadowed other than at the rater’s discretion. Variability would be reduced if discrete guidelines were implemented as to when incomplete CFPs should and should not be analyzed.

We also investigated how well the program measured CFVR. Despite some of the PV values being significantly different, 4/6 animals had differences less than 5% between the two methods. The program significantly underestimated PV for Animal 6 under both baseline and hyperemic conditions, yet the CFVR was nearly equal to the manual assessment. This highlights the importance of consistency when assessing TTDE CFPs. While the program did at times underestimate PV, the automated nature of a program like this will inherently produce consistent measurements across all flow patterns and subjects compared to the subjectivity of individual raters and their potential unconscious bias.

Finally, we introduced the aspect of dissecting and defining CFP cycles further into four phases based on the clear changes in slope during systole and diastole. There appears to be a lack of consistency in CFP nomenclature so we felt it necessary to define these measurements based on our and other investigators interpretations of TTDE CFP cycles. There have been a number of recent studies published on human CFPs suggesting that parameters other than PV, HR, VTI, and CFVR may be clinically diagnostic. Sezer *et al*. found that patients with T2DM, compared to patients without the disease, had a steeper deceleration of diastolic coronary flow^15^. Magagnin *et al*. similarly found in human CFPs that the diastolic slope was doubled in patients with left anterior descending coronary stenosis, connective tissue disease, and diabetes mellitus at baseline compared to normal^14^. Using our program, we found significant differences in time, velocity, and slope parameters between normal and diabetic mice especially under hyperemic conditions. And similar to the aforementioned studies, much of these differences were observed in the diastolic portion of the cardiac cycle. These differences detected by our program may highlight some aspects of early coronary microvascular remodeling that may otherwise go unnoticed in the traditional clinical setting. Future studies will investigate these parameters further to determine if they have any diagnostic or predictive value in determining the onset and progression of microvascular disease in diabetes.

### Limitations

We showed that our automated program was able to effectively reduce observer bias, reduce analysis time, and measure PV, HR, VTI, and CFVR with reasonable accuracy compared to manual analysis, especially in clear biphasic TTDE CFPs. There were still significant differences for some animals especially at baseline conditions and when measuring VTI. Future studies should aim to improve upon the algorithm for overlaying the CFP envelopes particularly when certain cycles are shadowed. Potentially incorporating a machine learning algorithm in conjunction with image processing techniques could lead to better envelope overlay and parameter selection.

Our study focused on a single Doppler ultrasound machine used in animal models. Creating a more flexible software that can conform to other ultrasound machine templates would be the next step in creating a comprehensive program. Additionally, there are subtle differences between human and murine CFPs, notably in the systolic portion of the cycle. The algorithm for selecting parameters in this region would likely need to be altered if the program were to be used on human subjects.

Lastly, our program is not entirely automated as the user is still required to manually export the raw video files, input scaling factors, and potentially alter the envelope threshold in the program. Even so, it is much quicker than manual analysis and similar to other non-coronary flow TTDE programs that have reduced time of analysis by 7.5-25 fold^14,20,22^. However, creating a fully automated program would reduce the analysis time even further.

## Conclusions

We developed a MATLAB program that can analyze murine PW Doppler CFP video files from the Vevo2100 Doppler ultrasound machine faster than manual analysis while reducing user bias. Building upon the limited number of previously published studies, our program supports raw exported AVI video files which can incorporate a larger number of flow pattern cycles for analysis than single still frame images. We also defined additional parameters of CFPs that may have useful diagnostic implications in the future.

## Electronic supplementary material

Supplementary Information

## Data Availability

No datasets were generated or analyzed during the current study.
