# Supplementary material for: Defining Coronary Flow Patterns: Comprehensive Automation of Transthoracic Doppler Coronary Blood Flow
Source: Sci Rep. 2018 Nov 22;8:17268. doi: 10.1038/s41598-018-35572-4 (PMC6250694; doi:10.1038/s41598-018-35572-4)
Supplement: Supplementary file 1 — Supplementary Information [file 41598_2018_35572_MOESM1_ESM.docx]

**Defining Coronary Flow Patterns: Comprehensive Automation of Transthoracic Doppler Coronary Blood Flow**

Ian L. Sunyecz^1^, Patricia E. McCallinhart^1^, Kishan U. Patel^1^, Michael R. McDermott^1^, Aaron J. Trask^1,2^

^1^Center for Cardiovascular Research

The Heart Center

The Research Institute at Nationwide Children’s Hospital

Columbus, OH

^2^Department of Pediatrics

The Ohio State University College of Medicine

Columbus, OH

Address for Correspondence:

Aaron J. Trask, PhD, FAHA

Center for Cardiovascular Research and The Heart Center

The Research Institute at Nationwide Children’s Hospital

Department of Pediatrics, The Ohio State University College of Medicine

700 Children’s Drive, WB4135

Columbus, Ohio 43205

Phone: 614.355.5760

Fax: 614.355.5725

Email: [aaron.trask@nationwidechildrens.org](mailto:aaron.trask@nationwidechildrens.org)

**Supplementary Methods**

*2. Region of Interest Extraction*PW Doppler Files

First, each frame of the video file was converted to grayscale using a built in MATLAB function. The pixel values were then averaged across each row and column. The immediate row above and the immediate columns to the left and right of the Doppler window region contained no image information and therefore had a mean pixel value of 0. Similarly, the row below the Doppler window region and ECG recording contained no image information and also had a mean pixel value of 0. These locations were used to crop each frame in the video file, which excluded the irrelevant information and kept only the Doppler window region and ECG recording.

Next, the individual frames were parsed together to create a single image containing the entire CFP sequence and ECG recording. Briefly, each consecutive frame in the video files contains a new amount of updated image information as well as information from the previous frames. When the Doppler sweeper reaches the right-most boundary, it resets back to the left boundary and overlays old image information with new image information. Taking the difference of consecutive frames leaves only the updated image information. The difference between consecutive frames for the entire sequence was calculated and used to identify when the sweeper reached the right-most boundary. These frames were stored and sequentially parsed together to create a single image containing the full Doppler CFP sequence and ECG recording

Finally, the Doppler CFP sequence and ECG recording were separately cropped and stored for analysis. The pixel values were averaged across each row and the horizontal zero-velocity baseline was identified as the maximum mean pixel value. The mean pixel value in the row immediately above the ECG recording contained no pixel information and was used to crop the ECG recording.

*3. Feature Extraction and Parameter Measurements*PW Doppler Files - Envelope Overlay:

The pixels for the full CFP and ECG sequence were converted to time and velocity values based on scaling parameters manually input by the user. The grayscale CFP sequence was filtered using a 2-D Gaussian filter with a sigma value of 3 and a spatial filtering domain. A linear morphological structuring element 5 pixels in length and 90 degrees from the horizontal axis was then created and used to dilate the CFP. The global threshold level was then calculated using a built in MATLAB function that utilizes Otsu’s method. The CFP sequence was converted to binary based on the global threshold value. Despite the preliminary filtering and thresholding, noise was still often present as small clusters of pixels above the Doppler flow pattern. To eliminate these areas, pixel clusters containing less than 100 pixels were automatically removed from the binary image. A final step was taken to remove noisy signals that persisted at the top of the Doppler window due to aliasing and/or non-specific blood flow picked up by the ultrasound probe. Noisy pixel clusters touching the top of the CFP window only were removed while all other pixels were preserved.

The binary image was then overlaid with the original grayscale CFP image to show the user the effective flow pattern envelope via the automated image processing techniques. This step was deemed crucial because parameters from the CFPs are extracted following this step. The envelope needs to precisely overlay the original Doppler CFPs in order to obtain accurate and precise measurements. Furthermore, each PW Doppler file acquired by a technician may not be identical. For example, contrast and brightness settings are often altered in order to obtain the clearest flow pattern. Moreover, the amount of noise often varies per echo based on probe penetration depth and tissue that is proximal to the probe relative to the artery. Therefore, creating an entirely automated algorithm to extract the envelope ideally for all PW Doppler CFP files would be challenging and potentially erroneous. Providing the user the ability to visualize and adjust the global thresholding level to better overlay the Doppler CFP envelope seemed to be an appropriate option while still being greatly efficient.

To isolate only the pixels that made up the CFP envelope and to create an envelope array, each column was scanned from the top of the image to the bottom. The first white pixel identified in each column was determined to be the envelope pixel. The same procedure was performed on the ECG recording after converting the image to binary. Using the pixel scaling measurements, the CFP envelope and ECG waveform rows and columns were mapped to their respective time and velocity values. Finally, a zero-phase 3rd order low-pass Butterworth filter was applied to the CFP envelope to smooth out the flow pattern

*PW Doppler Cycle Exclusion, Averaging, CFVR*:

Following parameter extraction for each cycle in the PW Doppler file, the PV values were automatically sorted into two bins. The smaller bin value was divided into the larger bin value to obtain a PV threshold. If the threshold value was less than or equal to 0.5, that indicated that one or more cycles were unlike the majority of cycles. Any cycle with a PV less than 50% of the average was excluded.

Despite filtering and smoothing the CFP envelopes, some parameters selected presented as outliers when compared to the overall average of the sequence. Any cycle that contained an outlier was also excluded from analysis. This was determined by calculating the average and +/- 2 standard deviation limits of each parameter. If an individual parameter from a CFP cycle fell outside of the high or low limit, the entire cycle was excluded. The remaining cycles were deemed representative of the entire CFP sequence and their parameters were averaged.

**Supplementary Figures and Tables**
